# Supplementary material for: Scalable High-Mobility Graphene/hBN Heterostructures
Source: ACS Appl Mater Interfaces. 2023 Jul 31;15(31):37794–801. doi: 10.1021/acsami.3c06120 (PMC10416142; doi:10.1021/acsami.3c06120)
Supplement: Supplementary file 1 — am3c06120_si_001.pdf [file am3c06120_si_001.pdf]

Supporting information

Scalable high-mobility graphene/hBN heterostructures

Leonardo Martini[1,], Vaidotas Mišeikis[1,2], David Esteban[3], Jon Azpeitia[3], Sergio Pezzini[4], Paolo Paletti[1,2], Michał W. Ochapski[1,2], Domenica Convertino[1], Mar Garcia Hernandez[3], Ignacio Jimenez[3], Camilla Coletti[1,2,*]*

1. *Center for Nanotechnology Innovation @NEST, Istituto Italiano di Tecnologia, Piazza San Silvestro 12, 56127, Pisa, Italy*
2. *Graphene Labs, Istituto Italiano di Tecnologia, Via Morego 30, I-16163 Genova, Italy*
3. *Instituto de Ciencia de Materiales de Madrid, Consejo Superior de Investigaciones Científicas, E-28049 Madrid, Spain*
4. *NEST, Istituto Nanoscienze-CNR and Scuola Normale Superiore, Piazza San Silvestro 12, 56127, Pisa, Italy*

leonardo.martini@iit.it, camilla.coletti@iit.it

a) BN and BNC films

BNC films with a thickness of 10 nm and with crystalline orientation parallel to that of the target substrate were synthesized using B_4C as precursor and their morphology and spectroscopic features were compared to those of the BN films. AFM analysis, reported in Figure S1, reveals a rougher morphology for the BNC films (root mean square (RMS) roughness of 950 pm) with respect to BN films of the same thickness (RMS roughness 935 nm). Raman spectroscopy indicates that both BNC films present a much lower E_{2g} Raman peak, compared to the BN film, suggesting a lower crystalline quality.

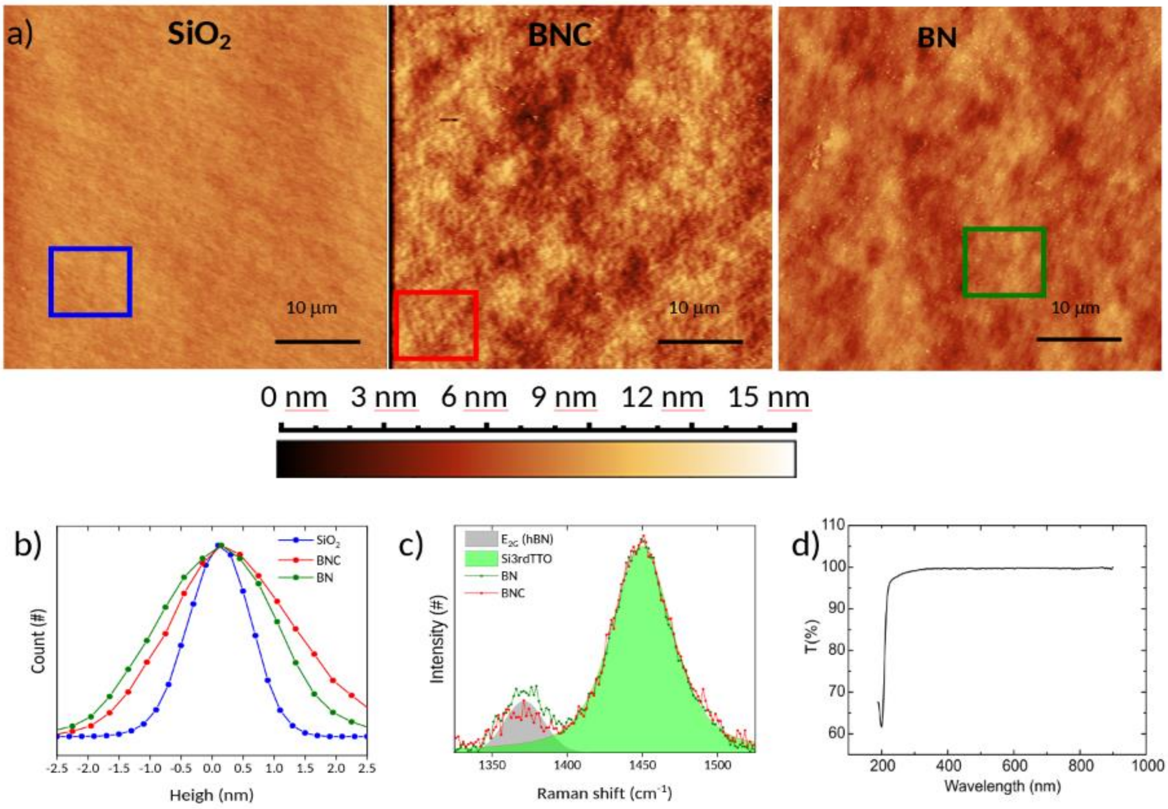

Figure S 1 a) AFM micrographs of representative BN and BCN samples, compared with the bare SiO_2/Si growth substrate. b) Height distribution of the different substrates, over a 10x10 μm^2 area. c) Representative Raman spectra for the BN and BNC samples. Both samples show a E_{2g}(hBN) peak at ~1370 cm^{-1}, with similar FWHM ~ 37 cm^{-1}. The E_{2g}(hBN) peak shows lower intensity for the BNC sample. d) UV-Vis spectrum of hBN grown on sapphire. The hBN thickness is determined applying the Lambert Beer relation ($I = I_0 e^{-\alpha d}$, where I_0 is the initial intensity, I is the intensity at 200 nm, α is the absorption coefficient at 200 nm and d is the thickness of the film)

The FWHM of the 2D Raman peak of graphene single-crystals transferred on SiO_2 and BN are comparable (i.e., 25 and 23 cm^{-1}, respectively), while larger values are retrieved for BNC substrates (i.e., 30 cm^{-1}). Raman correlation plots suggest that graphene doping and strain are comparable on BN and SiO_2, while BNC performs worse as a substrate.

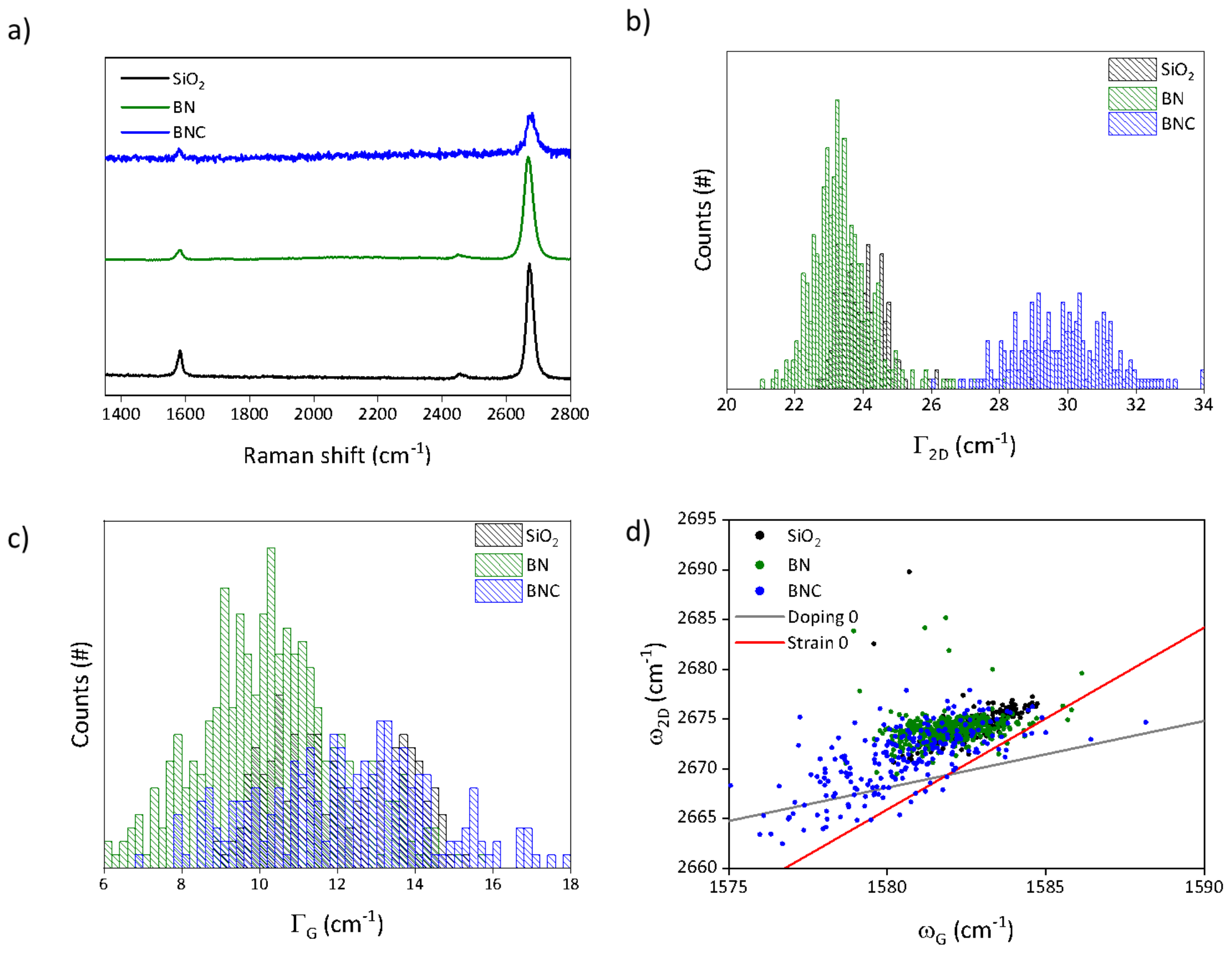

Figure S 2 a) Comparison of representative Raman spectra of graphene transferred on SiO_2 (black), 10nm-thick BN in green, and 10nm-thick BNC in blue. b) Distribution of the 2D-peak FWHM. The average value for the graphene transferred on SiO_2 and BN is of 25 and 23 cm^{-1} respectively, while on BNC is of 30 cm^{-1}. c) The distribution of the G-peak FWHM show no relevant change between the different substrates, averaging around 13 cm^{-1} for both BNC and SiO_2. d) Correlation plot between the position of the G-peak and 2D-peak; as reference, we show the evolution with strain for un-doped graphene (black), and with doping for the un-straied case (red).

b) Gate hysteresis and effect of annealing

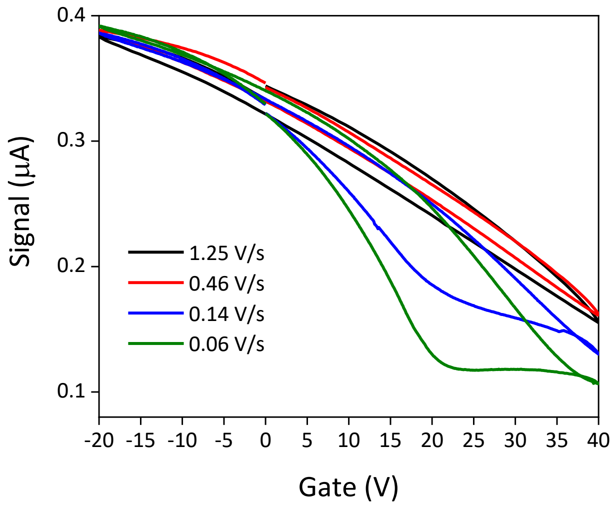

Figure S 3 Hysteresis dependence on the sweep rate: transfer curves on the same devices, performed at different gate sweeping speeds. The hysteresis increases at slow rate, indicatihng a relatively slow charge trapping dynamic.

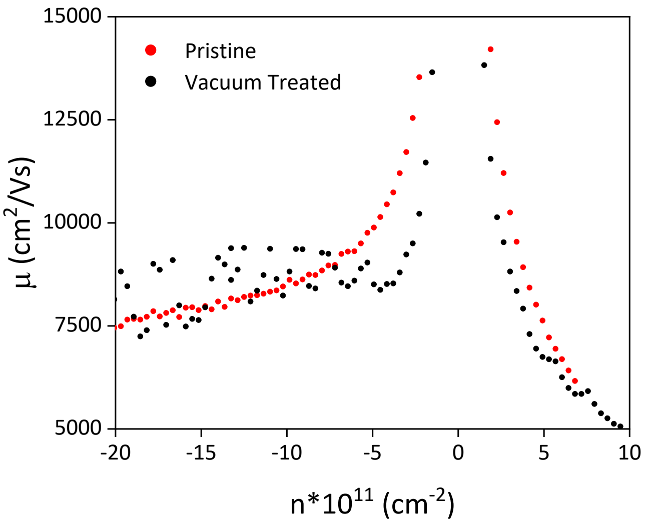

Figure S 4 S In red we report the mobility as function of the carrier concentration, obtained from a Hall measurement: carrier mobility at technological-relevant concentration of 10^{12} cm^{-2} are μ_e = 5000 cm^2/Vs and μ_h = 7500 cm^2/Vs for holes and electrons, respectively. The difference in the mobility with respect to the values reported in the main text is expected from the different assumption in the two methods [53]. In black we report the Hall mobility as function of the carrier concentration for the same device after the vacuum treatment: we do not observe significant changes in the mobility.

Hysteresis in the transfer curves is generally an unwanted behavior in electronic devices, which has to be solved in order to make devices suitable for commercial applications. Keeping the prepared sample in a commercial desiccator has proven to work well, even if the time needed to reduce the hysteresis to a level comparable with graphene on SiO_2/Si is of the order of several weeks. To accelerate this process, we tested high-vacuum thermal annealing on the graphene/hBN devices. We annealed several samples at different temperatures and for different times: low-temperature annealing shows some promising results, with a relevant reduction in the hysteresis as shown in Figure S5a for a 130 °C process. However, this kind of annealing tends to be slow. On the opposite, annealing at higher temperature tends to degrade graphene quality, as shown by the pinning in the electron branch of the transfer curve of the sample annealed at 300 °C, as shown in Figure S5b. The degradation of graphene is also proven by Raman, as the 2D-FWHM increases from 23 cm^{-1} to more than 35 cm^{-1}, as shown in Figure S5d. Also the 2D/G peak intensity ratio is reduced, as shown in Figure S5c.

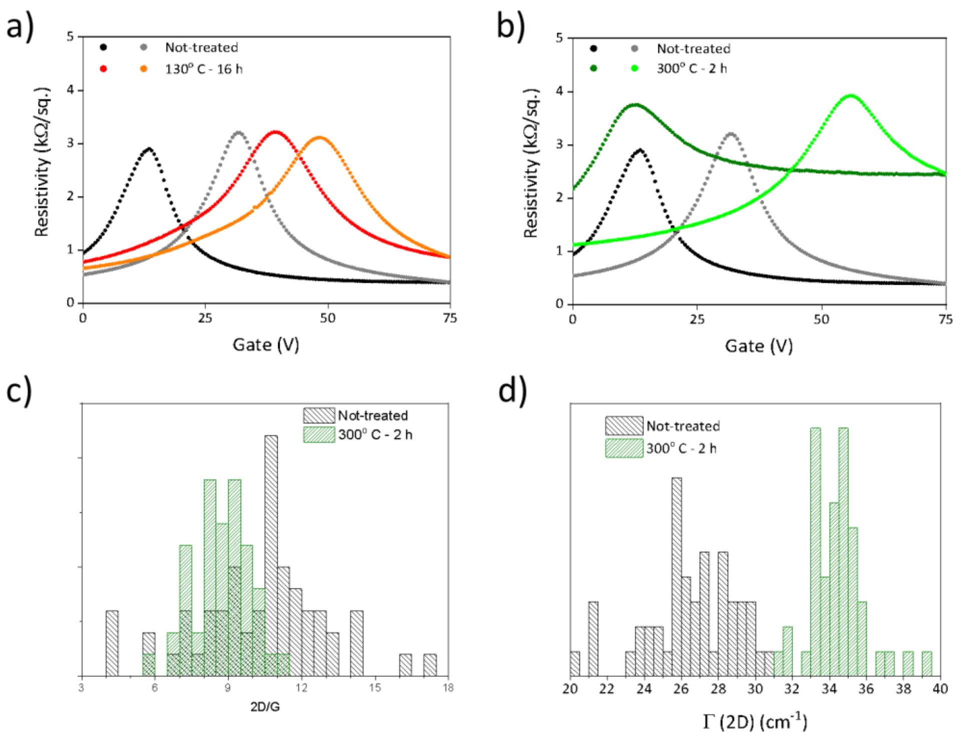

Figure S 5 A reduction of the hysteresis can be induced via ultra-high vacuum annealing at relatively low temperature (130 °C) for 16 hours. Annealing for shorter time didn't induce any reduction of the hysteresis, while increasing further the temperature led to a degradation of graphene. a-b) Transfer curve for comparable devices after fabrication and after 2 different annealing procedures: 130 °C – 16 hours in a) and 300 °C – 2 hours in b). c) 2D/G intensity ratio shows a reduction after the 300 °C annealing. d) The increase of the average 2D-FWHM after the 300 °C annealing also suggests a degradation of graphene.

c) Fully encapsulated graphene

To overcome the gating limitation at the untreated interface SiO_2/hBN in the system and prove the final achievable performance of this scalable graphene/hBN heterostructure, we implemented a top-gated device, using an exfoliated hBN flake as top-gate dielectric.

Through dry pick-up technique with exfoliated hBN, we transfer a portion of a graphene crystal[56], totally comparable with the one used in previous characterization, on IBAD-hBN. We use AFM and Raman (Figure S6) characterization to select a flat area for device fabrication. In Figure S7a we show a false-colour SEM image of the final device, we coloured in red the top-gate contact, in yellow the side contacts and in green the hBN/graphene heterostructure. The transfer curve obtained using the top-gate is reported in Figure S7b: the hysteresis is strongly reduced and the overall behaviour is compatible with similar devices made on commercially available SiO_2/Si substrates. The transfer curves are also more stable upon changing the gate sweeping speed.

Conversely, performing transfer curves on the same device using the back-gate (Figure S7c), we obtain the same hysteresis and overall behaviour of the fresh devices with exposed graphene. This result further indicates that the charge trapping responsible for the gate hysteresis takes place at the hBN/SiO_2 interface, unaffected by the dry transfer of the graphene with exfoliated hBN.

Finally, we test the overall performance of the fully encapsulated material, through Hall measurement. As shown in Figure S7d the carrier mobility reaches values >20,000 cm^2/Vs, remaining above 5 000 cm^2/Vs even at carrier concentration $>10^{12}$ cm^{-2}. The further improvement over the exposed graphene devices can be explained by the absence of environmental contamination on the surface of the graphene. This is also supported a reduction of the residual carrier density, reported in Figure S7d. The value of $n^* = 8.5x10^{10}$ cm^{-2} is half the

one of the exposed sample shown Figure 3e, in agreement with the increased values of carrier mobility.

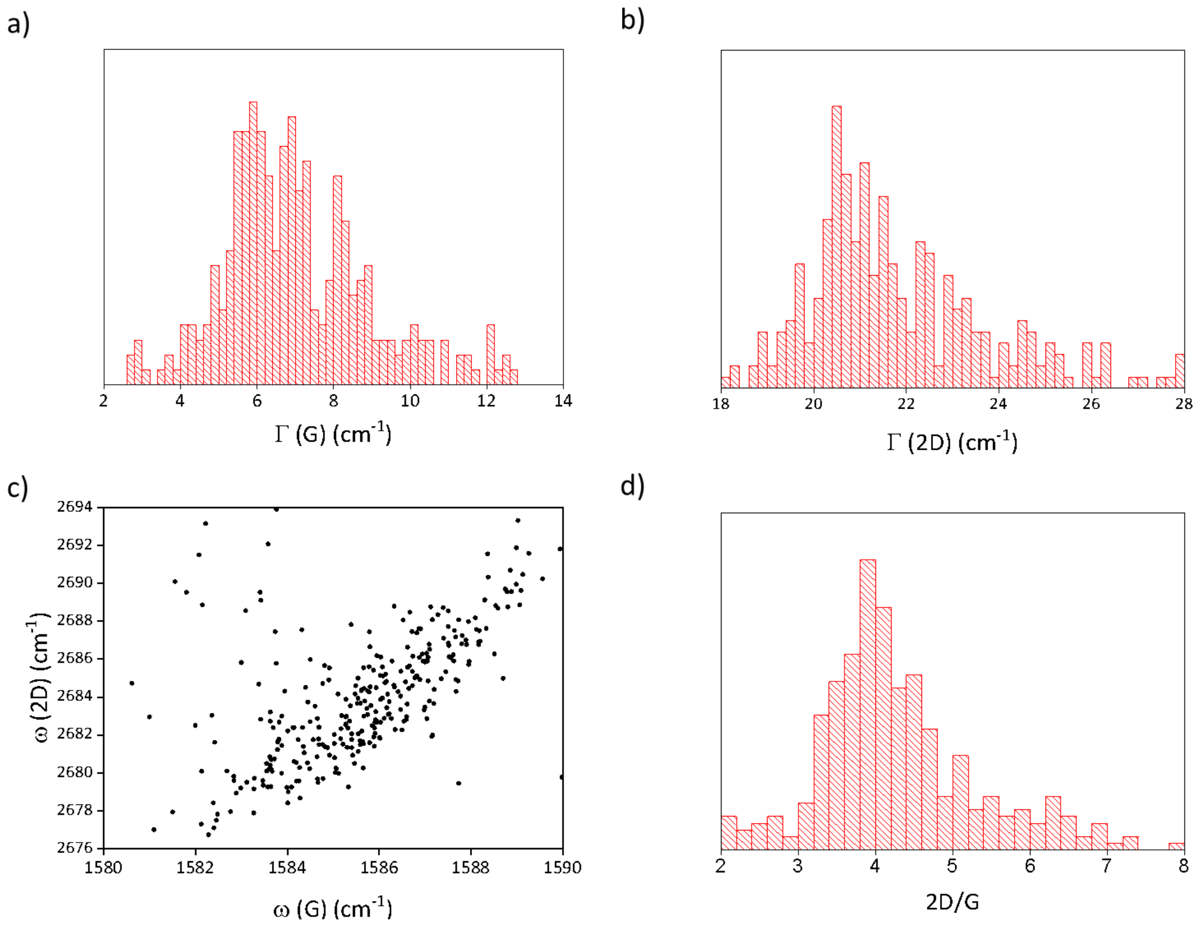

Figure S 6 Raman characteristics of the fully-encapsulate graphene sample. The G-peak FWHM in a), the 2D/G position correlation plot in c) and 2D/G intensity ration in d), suggest low doping level. b) The 2D FWHM, lower than the exposed graphene transferred both on SiO_2 and BN substrate, suggest sreduced strain variations. No D-peak has been observed in all the spectra acquired.

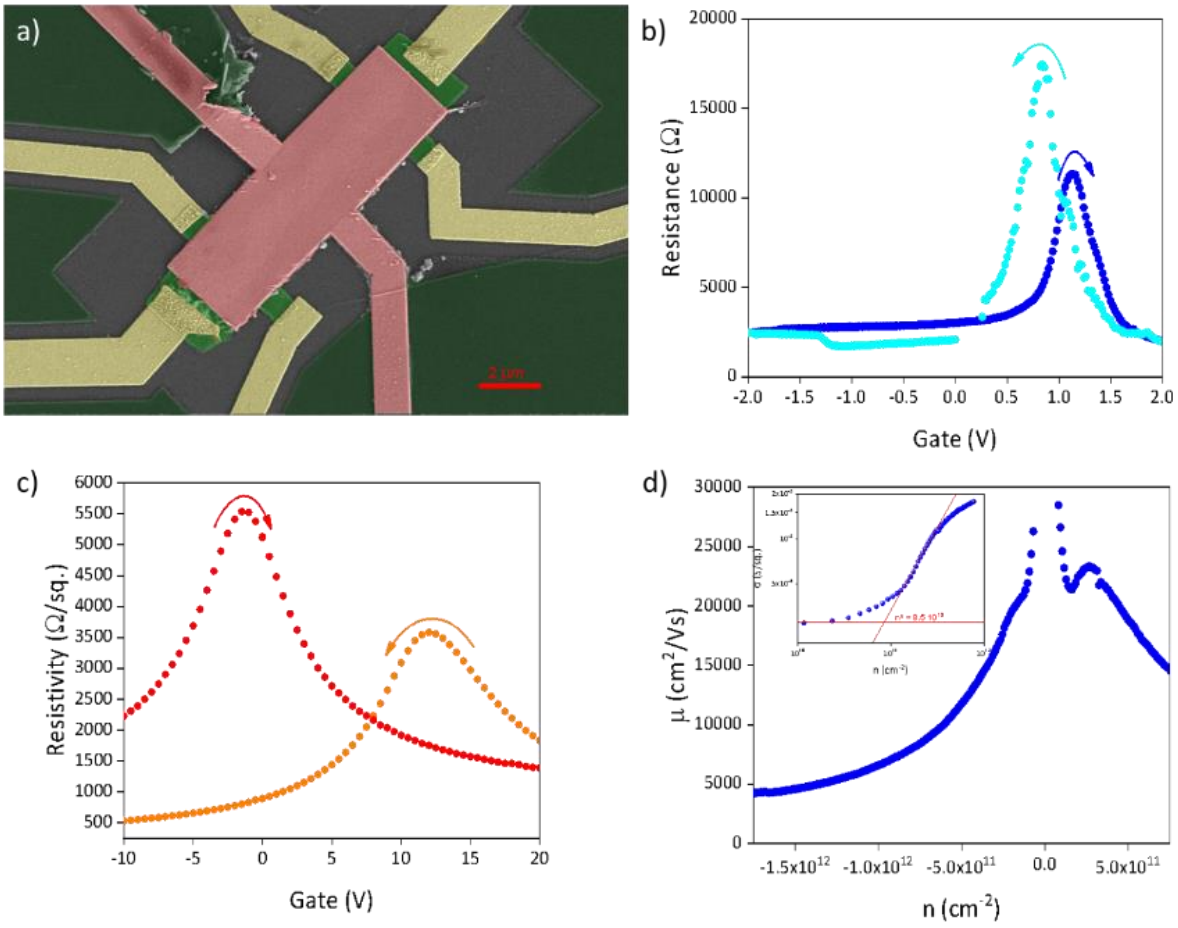

Figure S 7 a) false-color SEM image of the fully-encapsulated device: in dark green the unetched hBN/graphene, in yellow the metal side contacts, in red the top-gate and in green the device channel. The actual channel width is 6.6 µm and total length is 33 µm; several lateral contacts are presents each couple 8.7 µm apart, conferring an aspect ratio of 1.3 to the longitudinal measurements b) Transfer curve of the graphene on IBAD-hBN, using exfoliated hBN as the dielectric in a top-gated configuration: sweep from negative to positive (light blue) and from positive to negative (blue) gate values show reduced hysteresis. c) Transfer curve of the fully-encapsulated graphene, performed using the SiO_2/hBN back-gate. The hysteresis and difference between forward and backward sweep are present and comparable to the exposed-graphene devices. d) Moblility as function of carrier concentration for graphene on PVD-hBN top-encapsulated with exfoliated hBN. Inset: the double log plot of σ versus n indicates a residual carrier density of $n^* = 8.5x10^{10}$ cm^{-2}.
